# The functions of long non-coding RNAs in neural stem cell proliferation and differentiation

**DOI:** 10.1186/s13578-020-00435-x

**Published:** 2020-05-29

**Authors:** Yanfang Zhao, Hongliang Liu, Qili Zhang, Yuan Zhang

**Affiliations:** 1 Institute of Biomedical Research, Shandong Provincial Research Center for Bioinformatic Engineering and Technique, Zibo Key Laboratory of New Drug Development of Neurodegenerative Diseases, School for Life Science, Shandong University of Technology, Zibo, China; 2 Institute for Translational Medicine, Qingdao University, Qingdao, China

**Keywords:** Neural stem cells, LncRNA, Self-renewal, Neural differentiation, Neurogenesis, Gliogenesis, Neurological disorders

## Abstract

The capacities for neural stem cells (NSCs) self-renewal with differentiation are need to be precisely regulated for ensuring brain development and homeostasis. Recently, increasing number of studies have highlighted that long non-coding RNAs (lncRNAs) are associated with NSC fate determination during brain development stages. LncRNAs are a class of non-coding RNAs more than 200 nucleotides without protein-coding potential and function as novel critical regulators in multiple biological processes. However, the correlation between lncRNAs and NSC fate decision still need to be explored in-depth. In this review, we will summarize the roles and molecular mechanisms of lncRNAs focusing on NSCs self-renewal, neurogenesis and gliogenesis over the course of neural development, still more, dysregulation of lncRNAs in all stage of neural development have closely relationship with development disorders or glioma. In brief, lncRNAs may be explored as effective modulators in NSCs related neural development and novel biomarkers for diagnosis and prognosis of neurological disorders in the future.

## Introduction

In the central nervous system (CNS), stringently regulatory mechanism is essential for proper neural stem cells (NSCs) related development and functions. Recently, epigenetic modulators, especially long non-coding RNAs (lncRNAs) are found to be crucial for the maintenance of NSCs related biological activity. This review aims to introduce the functions and regulatory mechanism of lncRNAs in NSCs self-renewal and differentiated into neurons or/and glial cells.

## Neural stem cells

As a dynamic organ, vertebrate brain possesses the capacity of structural plasticity upon a variety of physiological, pathological and pharmacological stimuli owing to proliferation and differentiation ability of NSCs [1, 2]. Amazing huge number of neurons and glial cells constituting the cortex are generated from the differentiation of NSCs, which are able to self-renewal and major yield three forms of neural cells including neurons, astrocytes and oligodendrocytes in the brain [3], that the process of producing neurons and glial cells are termed neurogenesis and gliogenesis, respectively [4].

NSCs use symmetric divisions for adult neural precursor/progenitor cells (NPCs) amplification and asymmetric divisions for sequentially producing the right quantity of neurons and/or ultimately transition to gliogenesis sustaining postnatally [1, 4]. The largest NSCs niches throughout life are predominantly located in the subventricular zone (SVZ) nearby the lateral wall of the lateral ventricles and the sub-granular zone (SGZ) of hippocampus dentate gyrus (DG) [5]. The neuroblasts from NSCs in SVZ migrate along the rostral migratory stream (RMS) to the olfactory bulb (OB), where they terminally differentiate into local interneurons [6]; meanwhile, neuroblasts from NSCs in SGZ migrate short distances into the granule cell layer and mature into neurons, then integrate into functional circuit [7].

NSCs persist in the embryonic stage and in the specific area of the brain during adulthood [8]. However, it is still controversy whether adult neurogenesis occurs and even persist throughout lifetime. Some studies implied that proliferating progenitors and young neurons in the dentate gyrus (DG) sharply declined in the first year of life, only a few isolated young neurons were detected in the young and no young neurons were observed in DG [9, 10], whereas some others considered as hundreds of new neurons generated in each hippocampus/day in adult humans [11], and similar numbers of intermediate neural progenitors and thousands of immature neurons in the DG from young to older [12]. Undoubtedly, the generation of a certain number of neuronal progenitors from NSCs and then differentiation into neurons and/or glial cells are associated with brain development and changed in neurological disorders [13].

## LNCRNAs

As the rapid progress of next-generation sequencing technologies, a large amount of lncRNAs were discovered and identified as essential modulators in fundamental biological processes, although they were initially considered as “noise” of genome. LncRNAs are a sub-class of non-coding RNAs transcripts longer than 200 nucleotides with 5ʹm7G caps and 3ʹ poly (A) in tails, which are generated by RNA polymerase II but lack canonical protein-coding capacity [14, 15]. Clark et al found that majority of lncRNAs exhibit widely stabilities similar to that of mRNA, while the mean value of lncRNA half-life was 4.8 h that slightly less than the mean value of protein-coding transcripts (7.7 h) [16].

LncRNAs originate from various gene coding or non-coding locations including intergenic regions, introns, enhancers, promoters, exons, either with a partial overlap with protein-coding exons in both directions [17, 18]. They organize gene expression in the context by recruitment of regulatory proteins, modulation and modification of chromosomes at transcriptional level, controlling RNA splicing, acting as a “sponge” of miRNAs to regulate RNA degradation at post-transcriptional level and also participate in cytoplasm and nuclear trafficking or cell differentiation [18, 19].

**Fig. 1:**
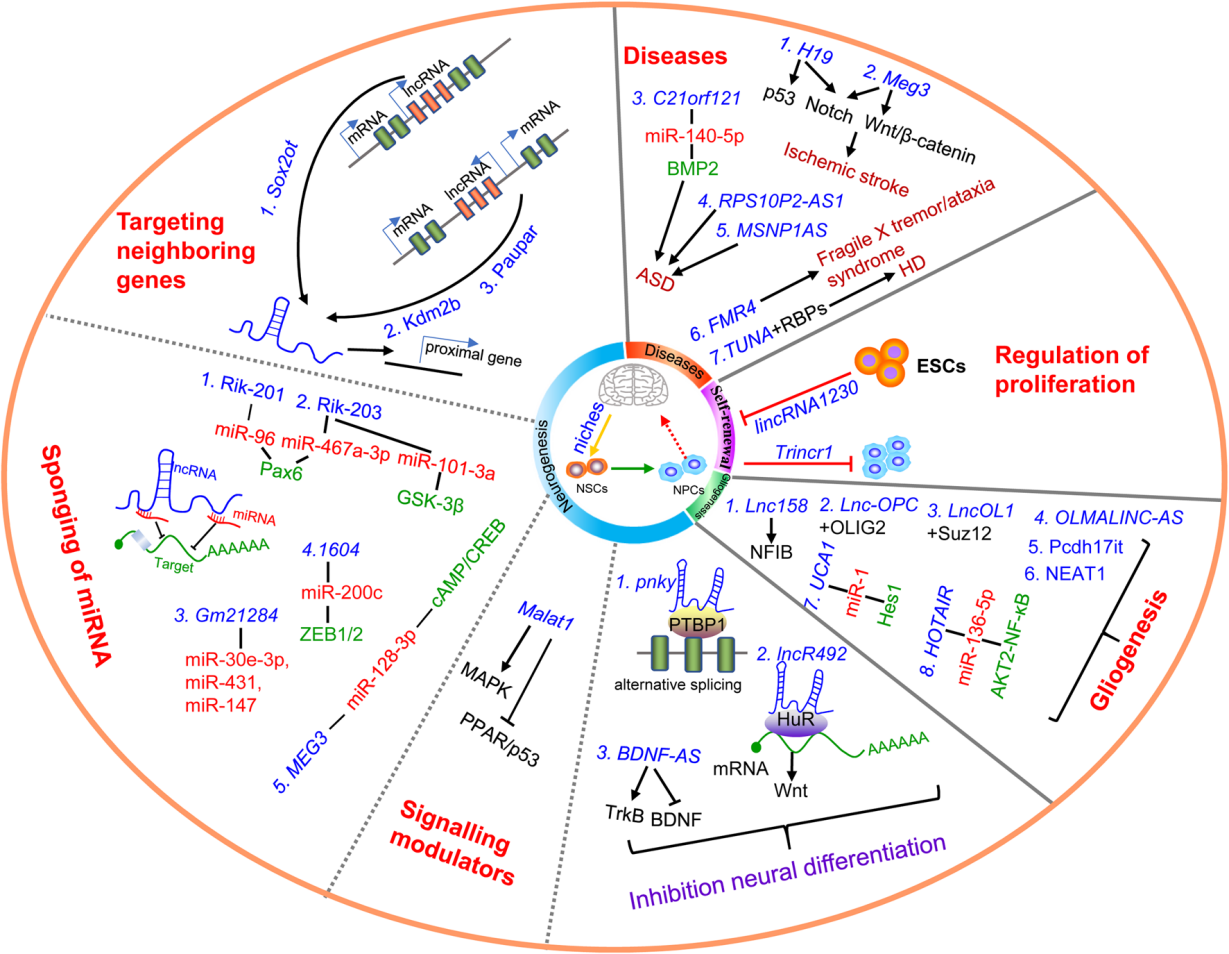
Schematic representation the regulatory networks of lncRNAs in major biology activities during brain developmental process. It was depicted in details that lncRNAs contribute to NSCs self-renewal, neurogenesis/neuronal differentiation, neurodevelopmental disorders and gliogenesis major emerging as sponging of miRNAs, crucial regulators in signaling pathway and targeting proximal gene expression

Accounting for 40% differentially expressed lncRNAs in human genome are specific to the brain, which involve in 4000–20,000 lncRNA genes [20]. LncRNAs have been reported be located in different brain cell types, such as neuron, glial cells and vascular cells, and playing crucial biofunction in the different brain cells [21]. In addition, lncRNAs are abundantly expressed in the particular NSCs generated regions of SVZ, DG or Striatum, which implies the crucial functional roles of lncRNAs in NSCs self-renewal, pluripotency, proliferation and differentiation [22–24]. The major goal of this article is to demonstrate the cell type-specific expression and functions of lncRNAs focusing on NSCs self-renewal, neurogenesis and gliogenesis over the course of neural development (Fig. 1).

## The effect of lncRNA on NSCs/NPCs self-renewal and proliferation capacity

As one type of multipotent cells, NSCs possess a significant capacity for proliferation and self-renewal, which are essential for maintenance of CNS homeostasis [25], moreover, they also can be derived from totipotent stem cells and various pluripotent cells in vitro [26, 27]. Although the underling regulatory mechanism still remains unknown, recently, some evidence implied that lncRNA may emerge as a modulator in NSCs self-renewal and proliferation (Table 1). For instance, overexpression of lncRNA *Trincr1* (TRIM71 interacting long noncoding RNA 1) suppressed the self-renewal of NPCs via restraining fibroblast growth factors (FGF)/extracellular signal regulated kinase (ERK) signaling pathway, which is essential for cell self-renewal [28, 29]. Furthermore, NPCs were increasingly transplanted from pluripotent stem cells for treatment neurological development disorders [26, 30]. *LincRNA1230* was able to markedly block mouse ESCs transformation into NPCs, mechanistically, it restrained the combination of WD repeat domain 5 (Wdr5) to the promoter regions of neural lineage-associated genes via reducing enrichment of the H3K4me3 (tri-methylation of histone3 lysine4) modification at these loci [31].

**Table 1:** The functional characterization of lncRNAs on neural related stem cells self-renewal

LncRNA name	Mechanism	Biological function	References
*Trincr1*	Restrain FGF/ERK signalling	Suppress NPCs self-renewal	[28, 29]
*LincRNA1230*	Interact with Wdr5	Block ESCs transformation into NPCS	[31]
*Linc00115*	Activated by TGF-β, miR-200 s/ZEB1, miR200s/ZNF596/EZH2/STAT3	Promote GSC self-renewal	[33]
*TUG1*	Activated by Notch, sponging of miR-145 and recruiting polycomb via YY1	Promote GSC self-renewal	[34]
*Linc00152*	miR-103a-3p/FEZF1 axis	Promote GSC proliferation	[35]
*ZNF281*	NF-κB1 pathway	Inhibit GSC self-renewal	[36]
*TALNEC2*	Regulated by E2F1, miR-21/miR-191	Promote GSC self-renewal	[37]
*Linc01198*	Enhancing the NEDD4-1-dependent inhibition of PTEN	Promote glioma proliferation	[38]

Glioblastoma stem-like cells (GSCs) exhibit the stemness properties of stem cell including self-renewing capability and multipotency [32], which progression and self-renewal are able to be modulated by lncRNAs. LncRNA *linc00115* activated by Transforming Growth Factor-β (TGF-β), lncRNA *TUG1* activated by notch receptor (Notch), *linc00152* and lncRNA *ZNF281* were newly identified lncRNAs that participated in controlling self-renewal and proliferation in GSCs via sponging of miR-200 s, sponging of miR-145, sponging of miR-103a-3p and targeting NF-κB1 signalling pathway, respectively [33–36]. Moreover, lncRNA tumor associated lncRNA expressed in chromosome 2 (*TALNEC2*) and linc*01198* were also found to promote self-renewal and progression of GSCs [37, 38]. Thus, lncRNA maybe a novel potential therapeutic strategy for glioblastoma therapy.

## The function of lncRNAs in neurogenesis/neural differentiation

Neurogenesis is a dynamic process associated with NSCs and NPCs differentiation into newborn neurons, which integrate into the local neural network in the mammalian CNS [39]. This process is a well-orchestrated sequence of complex biological and molecular event major including NPCs or NSCs proliferation and differentiation during pre- and post-natal brain development, which major occurs in SVZ and SGZ in DG of hippocampus [6, 40, 41].

Recently, as a partial of the mammalian genome, lncRNAs have emerged as crucial epigenetic regulatory elements implicated in NPCs or NSCs differentiation and neural development [40, 41]. Intricate expression regulation of lncRNAs in time and space is a crucial event in the developing CNS [42]. A quantity of lncRNAs are present abundantly in certain neurogenic cell-types or the specific functions in development and cellular identity in the nervous system [43, 44]. Furthermore, 13 lncRNAs temporally exhibiting neurogenic cell types specificity and hallmarks of RNA processing, have been noted in the purified neural cell types across consecutive time course overlap critical events in neurogenesis in Drosphila, proving that expressions of lncRNAs are highly dynamic and demarcates particular subpopulations within neurogenic cell types in CNS [45]. Interestingly, lncRNAs showed an adaptive function in the evolution of neurogenesis due to selective loss in the evolutionary time [46]. Actually, lncRNA was a key determinant in NSCs or NPCs during cell-fate determination, moreover, distinct lncRNA types are involved in the different situations of neurogenic precursor or stem cell differentiation. For instance, divergent lncRNAs have partiality for neuronal differentiation while sense downstream lncRNAs are more associated with astrocytic differentiation in NPCs or NSCs [47]. Moreover, lncRNAs can also participate in regulation the fate of NSC differentiation into glia and neurons in the physiology and pathological condition [48, 49]. In hence, it is still need to be discussed that lncRNAs how to play a role in neurogenesis/neuronal differentiation or gliogenesis (Table 2). Elaborating the underlying regulatory mechanism of neural differentiation is benefits for studying neural development or acquiring NSCs for diseases treatment.

**Table 2:** The roles of lncRNAs on NSCs differentiation/neurogenesis and oligodendrogenesis

LncRNA name	Mechanism	Biological function	References
*Sox2ot*	Link with Sox2, interact with YY1	Prohibit NSCs proliferation and advance neuronal differentiation	[54]
*RMST*	Target Sox2	Promote neurogenesis	[56]
*Kdm2b*	Bind with hnRNPAB and activate Kdm2b gene expression	Promote neurogenesis	[57]
*Paupar*	Bind with local genes-Pax6 and KAP1	Promote neurogenesis	[58, 59]
*Gm21284*	Interact with miR-30e-3p, miR-431 and miR-147	Inhibit NSCs proliferation while promote NSCs differentiation	[63]
*1604*	miR-200c/ZEB1/2 axis	Promote neural differentiation	[61]
*Rik*-*201*	Activated by C/EBPβ, miR-96/Sox6	Enhance neural differentiation	[64]
*Rik*-*203*	miR-467a-3p/Sox6, miR-101-3a/GSK-3β	Enhance neural differentiation	[64, 65]
*Malat1*	Activate ERK/MAPK, inhibit PPAR/p53	Promote neural differentiation	[67]
*Pnky*	Interact with PTBP1	Inhibit neural differentiation and neurogenesis	[3, 68]
*lncR492*	Interact with HuR and activate Wnt signalling	Inhibit neural differentiation	[69]
*BDNF*-*AS*	Targeting TrkB signaling pathway	Inhibit eNSCs-derived neurite outgrowth	[70]
*UCA1*	miR-1/Hes1	Promote NSCs differentiation to astrocyte but neuron	[48]
*OPC*	Regulated by OLIG2	Promote oligodendrogenesis	[87]
*lncOL1*	Form a complex with Suz12	Promote oligodendrogenesis	[90]
*lnc158*	Promote NFIB expression	Promote oligodendrogenesis	[91]
*Pcdh17it*		Oligodendrogenesis marker	[92]
*OLMALIN/*-*AS*		Regulate oligodendrocyte maturation	[93]

## The function of lncRNAs on promoting neuronal differentiation

LncRNAs meet a requirement for neuronal differentiation and are considered to be indispensable for neurogenesis. They are specific to the brain region, especially SVZ, DG or OB, highly expressed during neuronal differentiation, and always exert their functions via interacting with transcription factor or neighboring genes on the same chromosome, acting as competing endogenous RNA (ceRNA) of miRNA and target protein or emerging as key signaling pathway modulators.

### Effect on neighboring genes expression

LncRNAs are able to control neural development via influencing proximal protein-coding genes expressions. LncRNA Sox1 overlapping transcript (*Sox1ot*) and Sox2 overlapping transcript (*Sox2ot*) locating in nucleus are evolutionarily conserved lncRNAs that transcriptionally overlaps the Sox1 and Sox2, respectively, and considered as crucial modulators in the developing brain [50, 51]. Sox1 and Sox2 are transcription factors which associated with maintaining the stemness of pluripotent stem cells and NSCs [52]. *Sox1ot* and *Sox2ot* are highly expressed during neural development and link with Sox1 and Sox2 expression levels, respectively [53, 54]. Androgen receptor (AR),a transcription regulator in early embryonic stage, modulates lncRNA *Sox2ot* expression by interacting with Sox2 upstream DNA binding region at transcription level [55]. LncRNA *Sox2ot* prohibited NSCs proliferation and advanced neuronal differentiation by interacting with the transcriptional regulator YY1, which bind to CpG island in the Sox2 locus to suppress the expression of Sox2 [54]. LncRNA rhabdomyosarcoma 2-associated transcript (*RMST*) was considered to be indispensable for neurogenesis when its absence lead to more than 1000 genes differentially expression major via facilitating Sox2 binding the promoter regions and regulating the target genes [56]. In addition, lncRNA *Kdm2b* divergently transcribed from the same promoter bidirectional with *Kdm2b* and dispersed chromatin environment via binding hnRNPAB, then activated its nearby coding gene-Kdm2b expression for facilitating neuronal differentiation in early neurogenesis cortical projection neurons [57]. LncRNA *Paupar* divergently transcription from upstream of Pax6 participates in regulation of neural differentiation and OB neurogenesis via binding with local genes-Pax6 and KAP1 in *cis*-manner, as well as modulation the activity of various transcriptional regulatory elements on different chromosomes distinguishing from its synthesis locus and alteration of chromatin occupancy and H3K9me3 deposition [58, 59].

### Acting as ceRNA of miRNA

MiRNAs as a group of short non-coding RNAs (approximately ~ 22 nucleotides long) suppressing coding gene translation are abundant in the nervous system and participate in all stage of neural differentiation during brain development [60]. LncRNAs could participate in neural development via emerging as ceRNA of miRNA and indirectly regulate gene expression in the cytoplasm [61, 62]. Several differentially expressed lncRNAs interacting with miR-30e-3p, miR-431 and miR-147 were determined by microarray analysis in hippocampal pool. Among these lncRNAs, *Gm21284* was identified by function as a ceRNA to enhance the proportion of CHAT-positive cells during NSCs differentiation [63]. LncRNA *1604* silencing suppressed neural differentiation through acting as sponge of miR-200c to regulate the key transcription factors zinc finger E-box binding homeobox 1/2 (ZEB1/2) [61]. Moreover, lncRNA transcript could generate several variants to execute functions in neurogenesis. LncRNA C130071C03 Riken variants-*Rik*-*201* and *Rik*-*203* were also considered as modulators in the developing brain via being activated by neurogenesis related transcript protein CCAAT/enhancer-binding protein β (C/EBPβ). Suppression of *Rik*-*201* and *Rik*-*203* restrained neural differentiation via acting as ceRNAs of miR-96 and miR-467a-3p, respectively, which deinhibition restricted the expression of neural differentiation-related gene Sox6 [64]. Sevoflurane was also reported to attenuating the expression of lncRNA *Rik*-*203* that resulted in the release of miR-101-3a but lessening Glycogen Synthase Kinase-3β (GSK-3β) level, ultimately inhibited neural differentiation [65]. Furthermore, miR-128-3p is abundantly expressed in brain and emerges as a key modulator in neural differentiation, which overexpression prohibited neuron but enhanced gliocytes differentiation. LncRNA *MEG3* participated in promotion of neuron differentiation via emerging as a negative modulator of miR-128-3p while elevated by the cAMP/response element-binding protein (CREB) pathway [66].

### Emerging as key signalling pathway modulators

LncRNAs could also contribute to neural differentiation emerging as a pivotal member of signaling pathway. Neurite outgrowth is a core event in early neuronal differentiation and regeneration stage. The lncRNA Metastasis-associated lung adenocarcinoma transcript1 (*Malat1*) was indispensable for neurite growth. Knockdown of *Malat1* blocked neurite outgrowth but advanced cell death via suppression Mitogen-Activated Protein Kinase (MAPK) signaling pathway comparable with stimulation of Peroxisome proliferator-activated receptor (PPAR) and p53 signalling pathway [67].

## The role of lncRNAs on repressing neuronal differentiation

Compare to the above lncRNAs which be highly expressed and promoted neuronal differentiation, some other neuronal lncRNAs were revealed to be down-regulated in CNS and blocked neuronal differentiation. LncRNA *Pnky*, being considered as the first known neuronal development inhibitor as its expression was decreased when V-SVZ NSCs differentiation into neuronal cells, forming a complex with splicing factor and RNA-binding protein (RBP)-polypyrimidine tract-binding protein (PTBP1) participated in regulation of NSCs differentiation to neurons via controlling alternative splicing. Knockdown of either *pnky* or PTBP1 expression could strengthen neurogenesis, which both elicited a splicing program in cultured post-natal V-SVZ NSCs to mature neurons [3, 68]. Maria et al discovered the lncRNA *lncR492* as a lineage-specific inhibitor of neuroectodermal differentiation through interaction with mRNA binding protein HuR and activation of Wnt signaling pathway [69]. Furthermore, the enhanced expression of lncRNA brain derived neurotrophic factor antisense (*BDNF*-*AS*), which is an antisense RNA that inhibition of BDNF expression in neural growth, was able to inhibit neurite growth in ketamine treated mouse embryonic NSC-derived neurons via activating potassium uptake system protein (TrkB) signaling pathway [70].

## The effect of lncRNA on neurodevelopmental disorder via targeting NSCs/NPCs proliferation and differentiation

Addition to be as critical determinant for neuronal differentiation or neurogenesis in NSCs or NPCs, lncRNAs are also acting as pivotal regulatory molecules in several neurodevelopmental related diseases including Huntington’s disease (HD) [71], Autism spectrum disorder (ASD) [72], Angelman syndrome (AS) [73], vascular disorders induced ischemic stroke [74, 75]. LncRNA Tcl1 Upstream Neuron-Associated lincRNA (*TUNA*) was found to be associated with HD, which function for maintenance of pluripotency and neural differentiation by interaction with three RBPs [71]. Furthermore, lncRNA *FMR4* originating from Fragile X mental retardation 1 (FMR1) locus, which aberrant expansion causes autism [76], was able to improve hNPCs development, furthermore, dysregulation of *FMR4* contributed to the pathophysiology Fragile X syndrome and/or Fragile X tremor/ataxia syndrome [77]. In addition, lncRNA ribosomal protein S10 pseudogene 2 anti-sense 1 (*RPS10P2*-*AS1*), moesin pseudogene 1antisense (*MSNP1AS*) and *FMR4* were identified that contributed to another neurodevelopmental disorder-Autism spectrum disorder (ASD) risk [72, 77–79]. The expression of *RPS10P2*-*AS1* was elevated in postmortem temporal cortex of patients with ASD as well as in NPCs upon to ASD-associated diesel particular matter, which implied the close relationship between RPS10P2 with ASD risk [78]. LncRNA *MSNP1AS* expression was elevated in the postmortem cerebral cortex of individuals with ASD, which was mimicked by overexpression of *MSNP1AS* in human NPCs reduced neurite number and neurite length by disrupting moesin protein level, when knockdown of *MSNP1AS* blocked 318 genes expression, most of which participating in chromatin organization and immune response [72, 79]. Moreover, lncRNA *C21orf121* overexpression promoted conversion of stem cells from human exfoliated deciduous teeth into neuronal cells via acting as ceRNA of miR-140-5p to regulate BMP2 expression, which may provide a new therapeutic tool for ASD [80] (Table 3).

**Table 3:** The major roles of lncRNAs in neurodevelopmental disorders

LncRNA name	Mechanism	Biological function	Disease	References
*TUNA*	Interact with RBPs	Promote neural differentiation	HD	[71]
*FMR4*	Derived from FMR1 locus	Promote hNPCs proliferation	Fragile X syndrome	[77]
*RPS10P2*-*AS1*	Interact with RPS10P2		ASD	[78]
*MSNP1AS*	Regulate chromatin organization and immune response related gene	Inhibit neural differentiation	ASD	[72, 79]
*C21orf121*	miR-140-5p/BMP2	Promote neurogenesis	ASD	[80]
*H19*	p53/Notch1 pathway	Block neurogenesis	Ischemic stroke	[74]
*Meg3*	Notch or Wnt/β-catenin signaling pathway, miR-128-3p/ATRA/cAMP/CREB axis	Promote neurogenesis/neural differentiation	Ischemia-reperfusion injury	[75, 82]
*NEAT1*	Associated with Wnt signaling	Promote oligodendrogenesis	Schizophrenia	[94]
*HOTAIR*	miR-136-5p/AKT2-NF-κB		Demyelination	[95]

Furthermore, lncRNAs are involved in angiogenesis that NPCs in SVZ and SGZ migration to ischemic zone for restoration of blood supply after ischemic stroke [81]. One of the earliest identified lncRNA *H19* executed a negative function in chronic regeneration to inhibit neurogenesis process after ischemia stroke via inhibition p53/Notch1 signalling pathway [74]. Similarly, lncRNA *Meg3* also played a reversely effect on brain injury recovery and its absence improved nervous tissue impairment and promoted angiogenesis by triggering Notch pathway and Wnt/β-catenin signaling pathway [75, 82].

## The role of lncRNAs in modulation of gliogenesis

As well known, except for neurons, NSCs can gradually alter their characteristics to generate astrocytes and oligodendrocytes in the CNS, which termed as “gliogenesis” [83, 84]. At initial phase of cortical development, NSCs or NPCs sequentially produce deep layer neurons followed by surficial layer neurons; at later phase, NSCs suspend neurogenesis and shift to gliogenesis to gain gliogenic competence [84, 85]. The timing of NSCs transition from neurogenesis to gliogenesis must be stringently controlled to ensure proper cortical development [84, 86]. Several lncRNAs are considered as crucial modulators during neuronal-glial fate specification and oligodendrocyte lineage maturation. LncRNA human urothelial carcinoma associated 1 (*UCA1*) was able to decide the differentiation direction of NSCs, when *UCA1* silencing prohibited NSCs proliferation and differentiation to astrocyte but strengthen to neuron due to the enhancement of miR-1 expression but decrease expression of its target gene-Hes1 [48]. Moreover, some lncRNAs showed key functions for the fate of NSCs differentiate into glia and neurons exposure to hyperthermia [49]. Dong et al. screened 5000 lncRNAs to predict their roles in brain development and identified a highly conserved lncRNA-lnc-*OPC*. The expression of lnc-*OPC*, which upstream regulatory elements interaction with OLIG2, was dramatically enhanced in oligodendrocyte precursor cells (OPC) and contributed to OPC differentiation and oligodendrogenesis [87].

Myelination by oligodendrocytes is a vital event in the development and function of CNS and can be regulated by genetic and epigenetic factors including lncRNAs [88, 89]. The dynamic expression profiles of lncRNAs at different phases of oligodendrocyte growth were determined and then picked out a conserved chromatin-associated lncRNA-*lncOL1*. The gain of function of *lncOL1* enhanced precocious oligodendrocyte differentiation in neural development via forming a complex with a component of polycomb repressive complex 2 (Suz12), which is an oligodendrocyte maturation promoter [90]. Overexpression of *lnc158* in NSCs promoted several oligodendrocyte-related genes expressions and strengthened induction of oligodendrocyte lineage differentiation via positively modulation of an organ development regulatory factor-nuclear factor-IB (NFIB) [91]. In addition, an immature OL-specific lncRNA *Pcdh17it* was proved to be a novel biomarker for newborn immature OLs in the brain development [92]. Interestingly, lncRNA oligodendrocyte maturation-associated long intervening non-coding RNA (*OLMALINC*) has an identical expression type with its antisense counterpart, OLMALINC-AS, both abundantly expressed in the white matter of human frontal cortex and played vital roles in regulation of human oligodendrocyte maturation related genes [93]. In addition, oligodendrocyte-related abnormalities associated with developmental disorder including schizophrenia and demyelination are also regulated by lncRNAs [94, 95]. The expression levels of lncRNA *NEAT1* was reduced in the brain of patients with schizophrenia and loss of *NEAT1* influenced multiple oligodendrocytes cell differentiation related genes that caused population of oligodendrocytes-lineage cells diminishment during brain development [94]. LncRNA *HOTAIR* acting as ceRNA of miR-136-5p and AKT2-NF-κB was repressed and unbeneficial for repair impairment of cuprizone-induced demyelination [95]. Thus, lncRNAs indeed plays important functions in modulation of OL mature and oligodendrogenesis during brain development stage.

## Conclusion

Neural development related to NSCs/NPCs is considered as a huge complicate biological event. As advanced large-scale genome-wide sequencing analysis, more tissue-specific expression of lncRNAs were identified as essential modulators in fundamental neural developing biological processes. Most of their function remains to be explored, more novel lncRNAs and their molecular mechanisms remain to be found and probed in-depth yet. This review has described in detail the dramatically functional roles of lncRNAs in regulation of NSCs/NPCs self-renewal, proliferation and differentiation into neuron or glial cells, moreover, dysregulation of lncRNAs in all stage of neural development have closely relationship with development disorders or glioma. This suggest that lncRNAs have great potential to be applied in diagnosis, prognosis and treatment of neurodevelopmental disorders, still more, based on the features of their structural motifs, stability, easy-detectable and gene regulatory network, lncRNAs might be also employed as potential selection bio-markers for identifying or screening suitable NPCs/NPCs. With the deep-going study in the future, it will open a new era of lncRNA based NSCs proliferation and differentiation regulatory mode and neural development disorders therapy targets.

## Data Availability

Not applicable.

## Abbreviations

ASD: Autism spectrum disorder
AR: Androgen receptor
AS: Angelman syndrome
BDNF: Brain-derived neurotrophic factor
CREB: cAMP/response element-binding protein
C/EBPβ: CCAAT/enhancer-binding protein β
DG: Dentate gyrus
ERK: Extracellular signal regulated kinase
ESCs: Embryonic stem cells
FMR1: *FMR4* originating from Fragile X mental retardation 1
GSK-3β: Glycogen synthase kinase-3β
GSCs: Glioblastoma stem-like cells
HD: Huntington’s disease
LncRNAs: Long non-coding RNAs
Malat1: Metastasis-associated lung adenocarcinoma transcript1
MAPK: Mitogen-activated protein kinase
MEG3: Maternally expressed gene 3
MSNP1AS: Moesin pseudogene 1 antisense
NFIB: Nuclear factor-IB
NSCs: Neural stem cells
Notch: Notch receptor
NPCs: Neural precursor/progenitor cells
OB: Olfactory bulb
OPC: Oligodendrocyte precursor cells
OLMALINC: Oligodendrocyte maturation-associated long intervening non-coding RNA
PPAR: Peroxisome proliferator-activated receptor
PTBP1: RNA-binding protein (RBP)-polypyrimidine tract-binding protein
RMS: Rostral migratory stream
RMST: Rhabdomyosarcoma 2-associated transcript
RPS10P2-AS1: Ribosomal protein S10 pseudogene 2 anti-sense 1
SVZ: Subventricular zone
SGZ: Sub-granular zone
Sox1ot: Sox1 overlapping transcript
Sox2ot: Sox2 overlapping transcript
Suz12: Polycomb repressive complex 2
TALNEC2: Tumor associated lncRNA expressed in chromosome 2
Trincr1: TRIM71 interacting long noncoding RNA 1
TGF-β: Transforming growth factor-β
TUNA: Tcl1 upstream neuron-associated lincRNA
UCA1: Urothelial carcinoma associated 1
Wdr5: WD repeat domain 5
ZEB1/2: Zinc finger E-box binding homeobox 1/2
